# Dengue Viral Infection Induces Alteration of CD95 Expression in B Cell Subsets with Potential Involvement of Dengue Viral Non-Structural Protein 1

**DOI:** 10.3390/v17040541

**Published:** 2025-04-08

**Authors:** Siyu Wang, Premrutai Thitilertdecha, Ladawan Khowawisetsut, Theeraporn Maneesawat, Ampaiwan Chuansumrit, Kulkanya Chokephaibulkit, Kovit Pattanapanyasat, Nattawat Onlamoon

**Affiliations:** 1 Graduate Program in Immunology, Department of Immunology, Faculty of Medicine Siriraj Hospital, Mahidol University, Bangkok 10700, Thailand; wangsy_yn@163.com; 2 Siriraj Research Group in Immunobiology and Therapeutic Sciences, Faculty of Medicine Siriraj Hospital, Mahidol University, Bangkok 10700, Thailand; premrutai.thi@mahidol.ac.th (P.T.); theeraporn.man@mahidol.ac.th (T.M.); 3 Biomedical Research Incubator Unit, Research Group and Research Network Division, Research Department, Faculty of Medicine Siriraj Hospital, Mahidol University, Bangkok 10700, Thailand; kovit.pat@mahidol.ac.th; 4 Department of Parasitology, Faculty of Medicine Siriraj Hospital, Mahidol University, Bangkok 10700, Thailand; ladawan.kho@mahidol.ac.th; 5 Department of Pediatrics, Faculty of Medicine Ramathibodi Hospital, Mahidol University, Bangkok 10400, Thailand; ampaiwan.jua@mahidol.ac.th; 6 Department of Pediatrics, Faculty of Medicine Siriraj Hospital, Mahidol University, Bangkok 10700, Thailand; kulkanya.cho@mahidol.ac.th

**Keywords:** dengue, B cells, CD95 expression, apoptosis, NS1

## Abstract

Little is known about the regulation of B cell subpopulations in association with programmed cell death during dengue virus (DENV) infection. Therefore, blood samples from dengue-infected patients and healthy donors were obtained for B cell subset characterization and the analysis of pro-apoptotic CD95 expression in these cell subsets. The results showed that the activated memory (AM) subset in the patients remained unchanged compared to the healthy donors. In contrast, tissue memory (TM) and antibody-secreting cells (ASCs) were notably increased, whereas naïve cells and resting memory (RM) cells were considerably decreased. Although the ASCs maintained comparably high levels of CD95 expression in both groups, significantly increased percentages of CD95-expressing cells in the other B cell subsets were found in the patients. When B cells from the healthy donors were treated with DENV non-structural protein 1 (NS1), the results showed that the NS1 protein at 2 µg/mL could induce CD95 expression and the exposure of phosphatidylserine on the cell membrane in most B cell subsets, except for the RM. This study demonstrates that DENV infection could induce CD95 expression in both activated and resting B cell subsets in all patients. The results also suggest a potential mechanism of apoptotic regulation in B cell subsets through the increased CD95 expression caused by the interaction between the B cells and the NS1 protein.

## 1. Introduction

Dengue is a mosquito-borne disease caused by any of four antigenically distinct dengue virus (DENV) serotypes, including DENV-1, DENV-2, DENV-3 and DENV-4. It is the most prevalent arboviral infection worldwide, with diverse clinical manifestations ranging from mild fever (dengue fever, DF) to severe hemorrhagic fever (dengue hemorrhagic fever, DHF) and dengue shock syndrome (DSS). The prevalence is reported to be massive, with approximately 50–80 million infections occurring annually, leading to 500,000 cases of DHF and at least 12,000–24,000 fatal cases [1]. During the course of the infection, T cells and B cells are reported to be responsible for both protective immunity against DENV and the pathogenesis of the disease. While numerous studies have focused on T cell functions and alterations in dengue viral infection, limited information is available on B cells, and even less is known on B cell subsets. Furthermore, T cells has been reported to have almost absent responses and to not undergo significant apoptosis during DENV infection, indicating that T cells are resistant to DENV-induced apoptosis [2,3].

With respect to B cell responses against DENV, the primary reaction occurs when naïve B cells initially encounter the virus, inducing a proliferation of naïve B cells and differentiation into memory B cells and antibody-secreting cells (ASCs) to combat the existing infection. The secondary reaction occurs as a result of the re-exposure of dengue-specific memory B cells to the virus, leading to memory B cell clone division and expansion. To control excessive B cell immunity after their dynamic processes against the virus, control of the generation, survival, activation and maturation of the B cells is required via regulation of activation-induced cell death (AICD) [4,5]. Among the tumor necrosis factor receptor superfamily (TNFRSF) members, a pro-apoptotic surface marker (CD95/Fas receoptor, Fas/Apo-1/TNFRSF6) engaged to its ligand (CD95 ligan/Fas ligand, FasL/CD178) is crucial for maintenance in B cell homeostasis and peripheral immune tolerance through activation and selection in the germinal center [6]. Furthermore, viral infection potentially leads to the apoptosis of the host cells, which also involves the CD95/CD95L pathway [7]. Only one study has been conducted to investigate B cells expressing CD95 in patients with severe secondary DENV infection, and it suggests that DENV infection can promote activation-induced B cell death and B cell turnover [8].

DENV nonstructural protein 1 (NS1) is known for its importance in viral replication and pathogenesis [9], as well as being a key element used for dengue diagnosis [10]. Although DENV antigens have been reported for apoptotic induction through several mechanisms [11,12,13,14], there are few studies focusing on apoptosis stimulated by the NS1 protein. Previous investigations have shown that antibodies against the NS1 protein were able to cross-react with noninfected endothelial cells, leading to apoptosis via nitric oxide production [15,16] and the caspase-dependent pathway [17]. On the contrary, the addition of the NS1 protein could inhibit apoptosis in dengue-infected patient sera-induced endothelial cells [17]. No study regarding NS1-induced apoptosis in B cells has been found.

Therefore, this study aimed to determine the alteration of B cell subsets, including naïve, resting memory, tissue memory, activated memory and ASCs, together with their changes in CD95 expression during the course of dengue viral infection. The tentative mechanism of B cell apoptosis directly induced by the NS1 protein through the CD95/95L pathway was also evaluated.

## 2. Materials and Methods

### 2.1. Study Population and Sample Collection

Twenty-two dengue-infected patients and twenty-six healthy volunteers were recruited from the Faculty of Medicine Siriraj Hospital and the Faculty of Medicine Ramathibodi Hospital, Mahidol University, Bangkok, Thailand. The patients were categorized into three groups, including dengue fever (DF), dengue hemorrhagic fever (DHF) and dengue shock syndrome (DSS), based on severity of illness according to the 1997 WHO classification of dengue infection, which is currently an acceptable guideline for clinical practice in Thailand. All subjects provided informed consent prior to participating in this study. The study was ethically approved by the Institutional Review Board of the Faculty of Medicine Siriraj Hospital, Mahidol University, Bangkok, Thailand [COA number: 522/2015]. Blood samples of the individual donors were collected into vacutainer tubes containing sodium citrate (BD Biosciences, San Jose, CA, USA) and immediately transported to the laboratory for sample processing.

### 2.2. Classification of Dengue Virus Serotypes

Isolated plasma from the blood samples of the dengue-infected patients was extracted for ribonucleic acid (RNA) using a QIAamp viral RNA extraction kit (Qiagen, Hilden, Germany) according to the manufacturer’s instructions. The samples were then subjected into a multiplex nested reverse transcription polymerase chain reaction (RT-PCR, Promega Corporation, Madison, WI, USA) in order to identify the dengue virus serotypes (i.e., DENV serotypes 1–4).

### 2.3. Immunofluorescence Staining and Analyses

For the phenotypic characterization of the B cell subsets and their CD95 expression, whole blood (100 µL) was stained with a combination of fluorochrome-conjugated monoclonal antibodies against various cell surface molecules, including anti-CD3 conjugated with allophycocyanin-yanine 7 (APC-Cy7), anti-CD14 conjugated with Brilliant Violet™ 570 (BV570), anti-CD19 conjugated with BV510, anti-CD20 conjugated with Alexa Fluor^®^ 700 (A700), anti-CD21 conjugated with allophycocyanin (APC), anti-CD27 conjugated with BV650, anti-CD38 conjugated with BV421 and anti-CD95 (Fas) conjugated with phycoerythrin-cyanine 7 (PE-Cy7), which were obtained from BioLegend, San Diego, CA, USA, as well as anti-CD45 conjugated with peridinin chlorophyll protein (PerCP), which was purchased from BD Biosciences, San Jose, CA, USA. The stained cells were incubated in the dark at room temperature for 15 min. Two milliliters of FACS™ lysing solution (BD Biosciences, San Jose, CA, USA) was then added into the mixture and incubated in the dark at room temperature for another 15 min before centrifugation at 1400 rpm and 25 °C for 5 min. The supernatant was discarded, and the cell pellets were resuspended in 500 µL of FACS™ lysing solution before adding 2 mL of PBS. The sample was centrifuged at 1400 rpm and 25 °C for 5 min. After centrifugation, the supernatant was discarded, and the cell pellets were resuspended in 300 µL of PBS before being subjected to a flow cytometer. Fluorescence minus one (FMO) controls were also employed for the determination of CD95-expressing cells.

### 2.4. Cell Induction by Dengue Viral Nonstructural Protein 1 (DENV-NS1)

Whole blood samples obtained from the healthy volunteers were used for peripheral blood mononuclear cell (PBMC) isolation. The PBMCs were isolated from the collected blood samples by density gradient centrifugation over Ficoll-Paque (Histopaque 1077, Sigma-Aldrich, St. Louis, MO, USA) before lysing the red blood cells with ammonium–chloride–potassium (ACK) lysing buffer (Gibco, Grand Island, NY, USA) and washing with phosphate-buffered saline (PBS, Gibco, Grand Island, NY, USA). The isolated PBMCs were resuspended in a complete cell culture medium containig 10% fetal bovine serum (FBS, Gibco, Grand Island, NY, USA), 1% L-glutamine (Gibco, Grand Island, NY, USA) and 1% penicillin/streptomycin (Gibco, Grand Island, NY, USA). The counts of the isolated PBMCs were determined by the trypan blue exclusion method.

The PBMCs (1 × 10^6^ cells/mL in the complete medium) were cultured with different concentrations of purified recombinant DENV-NS1 (PROSPECT, Israel) at 0.1, 0.5, 1 and 2 µg/mL in a 24-well plate, while the culture medium alone was used as a control. The stock solution of DENV-NS1 was prepared at a concentration of 10 µg/mL in 1 × PBS containing 0.1% bovine serum albumin (BSA, Gibco, Grand Island, NY, USA) before being diluted into the required concentrations for cell induction. The samples were incubated overnight in a CO_2_ incubator at 37 °C and 5% CO_2_. After that, the samples were washed with 2% FBS (Gibco, Grand Island, NY, USA) in PBS prior to immunofluorescence staining for the CD95 expression and apoptosis analyses.

To investigate the CD95 expression on the DENV-NS1-treated cells, the cultured cells were washed twice with PBS and resuspended in PBS. The cells were then stained with a combination of CD95 PE, anti-CD3 APC-Cy7, anti-CD14 BV570, anti-CD19 BV510, anti-CD20 A700, anti-CD21 APC, anti-CD27 BV650, anti-CD38 BV421 and anti-CD45 PerCP and incubated in the dark at room temperature for 15 min before being washed with PBS and resuspending in 300 µL of PBS. The stained cells were then ready for the flow cytometric analysis.

For a study to detect the exposure of phosphatidylserine, the DENV-NS1-treated cells were washed twice with PBS and resuspended in Annexin V binding buffer (BioLegend, San Diego, CA, USA) at a concentration of 1 × 10^6^ cells/100 µL. The cells were then stained with a combination of annexin V PE, anti-CD3 APC-Cy7, anti-CD14 BV570, anti-CD19 BV510, anti-CD20 A700, anti-CD21 APC, anti-CD27 BV650, anti-CD38 BV421 and anti-CD45 PerCP and incubated in the dark at room temperature for 15 min before washing with annexin V binding buffer. After that, the stained cells were resuspended in 300 µL of annexin V binding buffer before being subjected to the flow cytometer.

### 2.5. Flow Cytometric Analysis

The LSRFortessa flow cytometer with FACSDiva software version 6.2 (BDB: San Jose, CA, USA) was used to analyze all prepared samples. The results of the B cell subsets and their CD95 expression, as well as apoptotic induction, were analyzed using FlowJo software version 10.10 (Tree Star: San Carlos, CA, USA).

### 2.6. Statistical Analysis

Statistical analysis was performed using GraphPad Prism^®^ software version 7.02 (GraphPad Software, Inc., La Jolla, CA, USA). The data were expressed as the means ± standard deviation (SD). Unpaired and paired *t*-tests were used to determine the differences in the mean values between the dengue-infected patients and healthy individuals, as well as among the non-induced and NS1-induced groups, respectively. *p*-values < 0.05 were considered as a statistical significance.

## 3. Results

### 3.1. Comparison of B Cell Subsets in Dengue-Infected Patients and Healthy Donors

**Figure 1:**
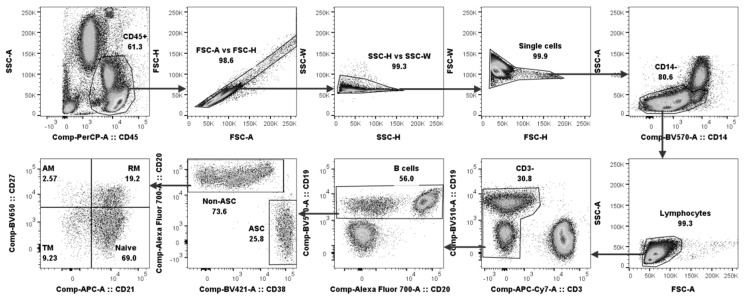
Representative gating strategy for B cell subset identification. Naïve, resting memory (RM), tissue memory (TM), activated memory (AM) and ASCs were characterized based on light-scattered properties and surface marker expressions including CD3, CD14, CD19, CD20, CD21, CD27, CD38 and CD45.

B cells play an important part in immune responses during the course of dengue viral infection; however, detailed information concerning changes in the B cell subpopulations is limited. The frequencies of B cell subsets including naïve, resting memory (RM), tissue memory (TM), activated memory (AM) and antibody-secreting cells (ASCs) in the dengue-infected patients were thus observed and compared to those in the healthy individuals. Forty-eight subjects were recruited in this study, and their demographic data and characteristics are summarized in Table 1. To identify the B cell subsets, the flow cytometric gating strategy was employed and is illustrated in Figure 1. The cells were first gated for hematopoietic cells (CD45^+^) before doublet discrimination via light-scattered properties. After that, monocytes (CD14^+^) were excluded, and only lymphocytes were selected based on their light-scattered properties. The non-T cells (CD3^−^) were gated and identified as total B cells from the CD19 and CD20 expression. The total B cells were then gated for ASCs (CD20^−^CD38^+^). The non-ASCs (CD20^+^) were further divided into four different B cell subpopulations based on the differential expression of CD21 and CD27, including naïve (CD21^+^CD27^−^), resting memory (CD21^+^CD27^+^), tissue memory (CD21^−^CD27^−^) and activated memory (CD21^−^CD27^+^).

**Figure 2:**
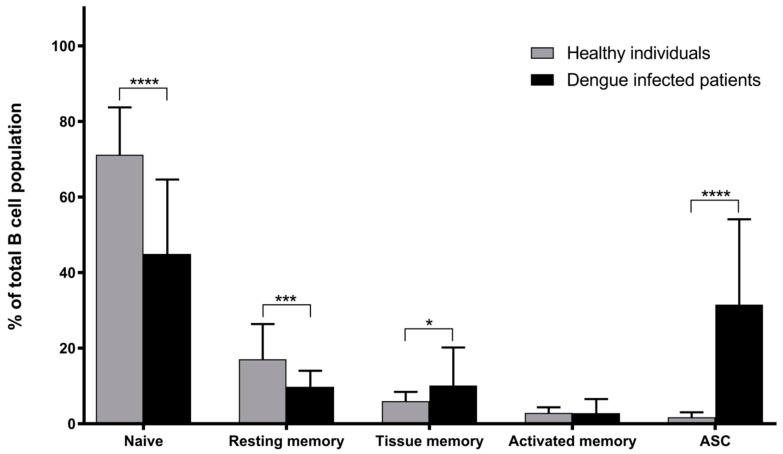
Percentages of B cell subsets in dengue-infected patients compared to healthy individuals. Changes in naïve, resting memory, tissue memory, activated memory B cells and ASCs were observed, and all data are presented as means ± SD (*n* = 22 for patient group, *n* = 26 for healthy group, * *p*-value < 0.05, *** *p*-value < 0.001 and **** *p*-value < 0.0001).

It is worth noting that although the samples from the patients were tested for DENV serotypes and classified by disease severity (i.e, DF and DHF), no significant difference was found among these patient groups which is in agreement with our previous study on the association between B cell subsets and the severity of DENV infection in pediatric patients [18]. Therefore, the results from both the DF and DHF patients were combined in this study. A comparison of the frequencies of the B cell subsets between the dengue-infected patients and healthy donors is demonstrated in Figure 2. The patient samples showed that the ASC level was markedly increased by 17.5-fold when compared to that of the healthy volunteers (31.5% versus 1.8%, respectively; *p*-value < 0.0001). Their tissue memory B cells were also slightly higher by 1.7-fold (10.1% versus 6.0%, respectively), whereas the naïve and resting memory B cells notably dropped by 1.6- (44.9% versus 70.6%, respectively) and 1.8-fold (10.1% versus 17.5%, respectively). However, no difference was observed in the activated memory B cells, which remained at low frequencies of approximately 3%.

### 3.2. Differences in CD95 Expression on B Cell Subsets Between Dengue-Infected Patients and Healthy Donors

**Figure 3:**
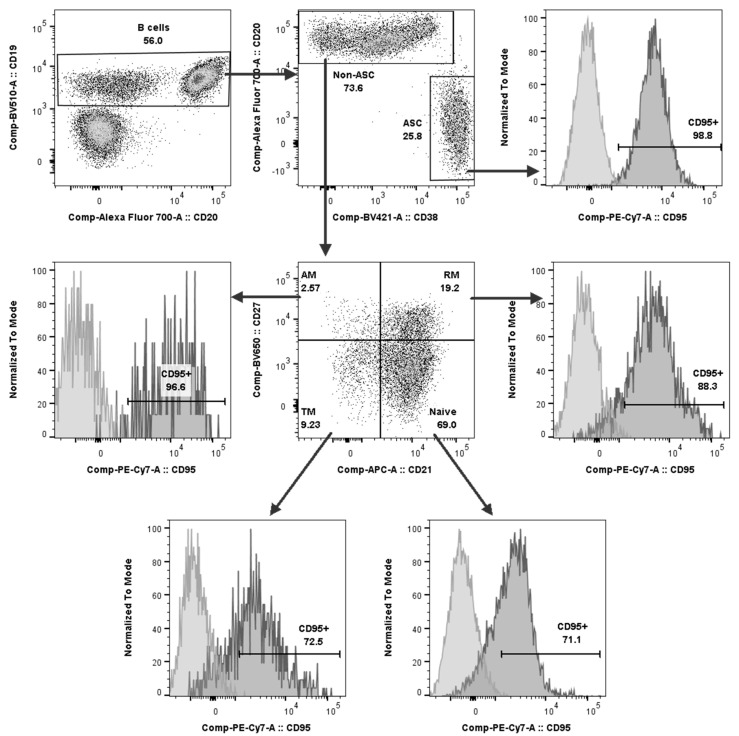
Representative gating strategy and histograms to identify CD95 expression in each B cell subpopulation. CD95 expressions in naïve, resting memory (RM), tissue memory (TM), activated memory (AM) B cells and ASCs were determined based on fluorescence minus one (FMO) controls.

To prevent the B cell responses from over-reacting after a viral infection, several mechanisms are involved as a negative feedback control, namely, programmed cell death. CD95 or Fas receptor is a pro-apoptotic marker regarded as a death receptor mediating apoptosis induction to maintain immune homeostasis. It is also considered an important surface molecule in the immune elimination of virus-infected cells. Therefore, changes in the CD95 expression on B cell subsets in the dengue-infected patients compared to healthy individuals were investigated in this study. Representative histograms of the CD95-expressing cells in each B cell subset are demonstrated in Figure 3, in which the CD95 expression on the B cells was evaluated based on the FMO controls.

**Figure 4:**
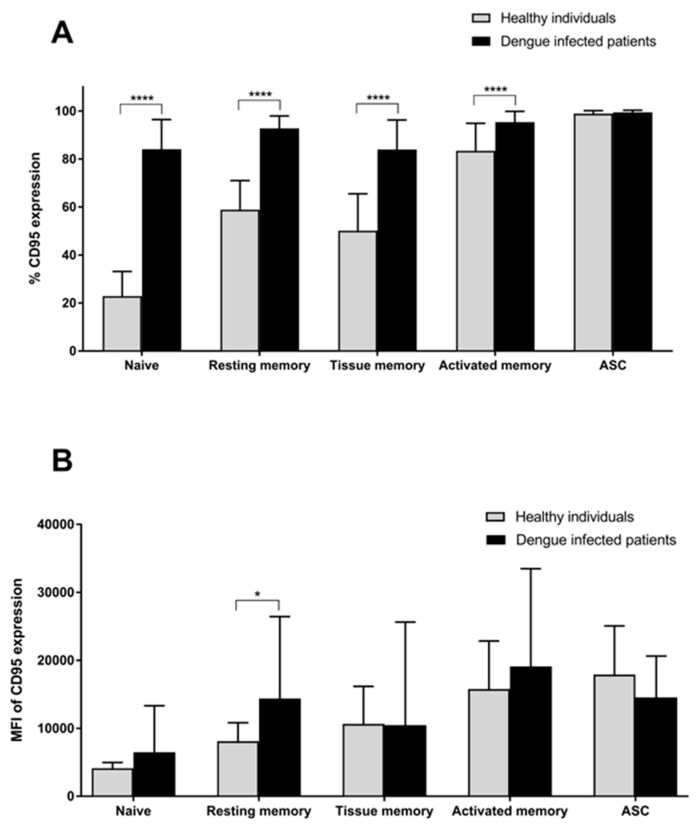
Changes in CD95-expressing B cells in dengue-infected patients compared to healthy individuals. (**A**) Percentages of CD95-expressing cells and (**B**) mean fluorescence intensities (MFI) of each B cell subpopulation (i.e., naïve, resting memory, tissue memory, activated memory B cells and ASCs) were observed. All data are presented as means ± SD (*n* = 22 for patient group, *n* = 26 for healthy group, * *p*-value < 0.05 and **** *p*-value < 0.0001).

The CD95-expressing B cells in each subset were examined in terms of both the percentages of the cells that expressed CD95 and the intensity of CD95 expression in the cell population (Figure 4A,B). The results from the healthy group showed that majorities of the ASCs and activated memory B cells expressed CD95 (99.1% and 84.7%, respectively). While CD95 expression was observed in most of the ASCs and activated memory B cells, other B cell subpopulations also expressed CD95. Approximately half of the resting memory (59.7%) and tissue memory B cell (50.8%) populations, as well as some of the naïve B cells (22.7%), expressed CD95. Similar results were observed in the dengue-infected patients. Interestingly, high frequencies of CD95-expressing cells in the naïve (84.1%), resting memory (92.8%) and tissue memory (84.0%) B cells were found, while similar levels to the healthy donors were observed for activated memory (95.4%) and ASCs (99.4%). Significant differences between the patient and healthy groups were found in every subset, except for the ASCs. The frequency of naïve B cells in the patient group was 3.7-fold greater than that in the healthy donors. The numbers of CD95-expressing cells in resting memory, tissue memory and activated memory B cells in the patients were also higher than in the healthy individuals by 1.6-, 1.7- and 1.1-fold, respectively. The mean fluorescence intensity (MFI) of CD95 expression was also assessed for the intensity of the CD95 moleules in all subpopulations (Figure 4B). Only the CD95 expression in the resting memory B cells was found to be considerably higher in the dengue-infected patients, by 1.8-fold, when compared to those in the healthy donors.

### 3.3. Apoptotic Induction by DENV NS1 Protein

During the acute phase of dengue viral infection, high levels of the viral protein NS1 can be dectected. The DENV-NS1 protein is also reported to be involved in the dengue viral life cycle and associated with severe clinical symptoms. However, there is no study concerning the mechanism of the NS1 protein on B cell apoptosis, even though DENV antigens are known for apoptotic induction [11,12,13,14]. This study thus conducted experiments using the NS1 protein at different concentrations (0.1, 0.5, 1 and 2 µg/mL) based on a previous study reporting NS1 levels in blood circulation during dengue infection [19] to identify its capabilities in apoptotic induction via CD95 and annexin V expression in B cell subsets.

**Figure 5:**
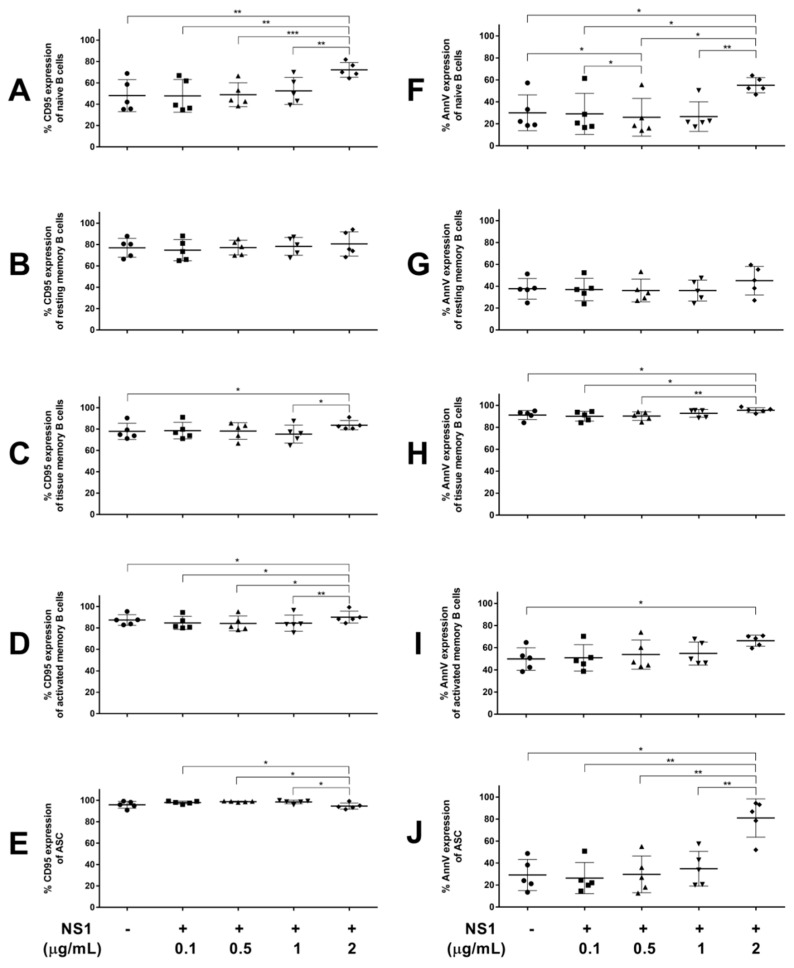
Effects of recombinant NS1 protein on CD95 and annexin V (AnnV) expression in different B cell subsets. Frequencies of CD95-expressing cells (**A**–**E**) and AnnV expressing cells (**F**–**J**) were determined in each B cell subpopulation, including naïve (**A**,**F**), resting memory (**B**,**G**), tissue memory (**C**,**H**), activated memory B cells (**D**,**I**) and ASCs (**E**,**J**), after treatment with NS1 protein at different concentrations (i.e., 0.1, 0.5, 1 and 2 µg/mL) compared to untreated cells. All data are presented as means ± SD (*n* = 5, * *p*-value < 0.05, ** *p*-value < 0.01 and *** *p*-value < 0.001).

The frequencies of CD95 expression in the B cell subpopulations after the incubation with the NS1 protein at various concentrations were determined (Figure 5A–E). High percentages of CD95-expressing cells were observed after overnight incubation in all B cell subsets, except for naïve cells. Of all concentrations, only the highest dose of NS1 at 2 µg/mL was able to induce CD95 expression in all B cell subsets, except for the resting memory B cells and ASCs. The percentages of CD95-expressing cells in the NS1-activated naïve, tissue memory and activated memory B cells were significantly greater by 1.5-, 1.1- and 1-fold when compared to the untreated controls. Nevertheless, the high levels of CD95 expression in the resting memory B cells and ASCs remained unchanged after NS1 induction (80.6% and 94.7%, respectively).

The role of the NS1 protein in apoptotic induction was also determined through the expression of an apoptotic marker (annexin V). The changes in the frequencies of annexin V-expressing cells in the B cell subpopulations after NS1 treatment at various concentrations were investigated, as seen in Figure 5F–J. Similar to the results of the CD95 expression, only the highest dose (2 µg/mL) of the NS1 protein was able to notably induce annexin V expression. The frequencies of annexin V-expressing cells in the NS1-treated naïve, tissue memory and activated memory B cells were significantly elevated by 1.8-, 1- and 1.3-fold when compared to the untreated controls. Interestingly, the ASCs after NS1 induction showed a remarkable level of annexin V expression, with 2.8-fold greater than in the untreated control, whereas the resting memory B cells remained unchanged.

## 4. Discussion

The differences in both quality and quantity of the primary and secondary antibody responses to dengue virus are partly due to responses mediated by different B cell subsets. While the primary response is a result of the first encounter between the naïve B cells and the virus, leading to an induction of naïve B cell proliferation and differentiation into ASCs and memory B cells, the secondary response is a result of repeated contact with dengue-specific memory B cells, leading to memory B cell clone division and expansion. This dynamic change in the B cell responses causes the alteration of B cell subsets during the course of dengue viral infection. In this study, the results showed an increased frequency of ASCs similar to those previously described [8,20,21,22]. An increased frequency of tissue memory B cells was also detected. In addition, a decrease in naïve B cells was observed, which is consistent with our previous report [18], together with decreased resting memory B cells, which is similar to a previous report on dengue patients in Northeast Brazil [8]. Taken together, the results demonstrate consistent observation of the B cell subset alterations, with a massive expansion of ASCs upon encountering the dengue virus.

B cell responses are dynamic processes followed by a contraction phase of responding cell subsets in order to control excessive immune responses. This phase is highly regulated by many mechanisms, including a programmed cell death or apoptosis [5]. CD95 expression can be upregulated in B cells following activation. This increased expression can make them more susceptible to apoptosis, serving as a feedback mechanism to control the extent of the immune response. Moreover, increased CD95 expression in B cells can contribute to abnormal B cell activation and survival, potentially leading to the production of autoantibodies. A previous study showed that B cells in individuals with severe secondary dengue infection significantly increased the expression of the pro-apoptotic marker CD95, or Fas receptor [8]. The significance of the CD95 expression in B cells was demonstrated by the induction of intracellular signaling, which leads to the apoptotic death of the B cells following the engagement of CD95 and its ligand [23]. Therefore, the alterations of different B cell subsets observed in this study may be regulated by the differential expression of CD95. In this study, most activated memory B cells and ASCs expressed CD95. While no difference in CD95-expressing ASCs was observed between the healthy subjects and dengue-infected patients, a relatively higher frequency of activated memory B cells expressing CD95 was observed in the patient group. These results confirm the long-established role of CD95 expression in activated B cells [24,25], suggesting that activated memory B cells and ASCs during acute dengue infection are susceptible to cell death regulated by CD95 interaction.

Although CD95 was reported to be expressed upon B cell activation, various amounts of naïve, resting memory and tissue memory B cells from the healthy subjects were found to express CD95. Interestingly, increased frequencies of these B cell subsets expressing CD95 were observed in the dengue-infected patients by approximately 80–90% of each B cell subset. A remarkable increase (approximately 4-fold) in the frequency of naïve B cells expressing CD95 was also observed in the dengue-infected patients when compared to the healthy subjects. No evidence of CD95 expression on naïve B cells during the course of dengue infection has been previously reported, but increased levels of subset-specific naïve B cells expressing CD95 were observed in systemic lupus erythematosus patients [26]. The presence of these CD95-expressing naïve B cells also exhibited hyporesponsiveness to toll-like receptor stimulation and decreased antibody-secreting abilities, suggesting a functional modification in this B cell subset. Along with functional changes, CD95-expressing B cells may undergo apoptosis due to the interaction of CD95 and its ligand, since increased CD95L was detected in the plasma of acute dengue-infected patients [27]. This then indicates the possibility that dengue infection induces altered functions and decreases in B cell subsets due to the expression of CD95.

Regarding a factor triggering the expression of CD95 during the course of dengue infection, a study showed that dengue virus infection could induce an increased level of CD95 in human umbilical vein endothelial cells [7]. Some studies have provided evidence supporting that dengue virus can infect B cells [28,29], whereas other studies have presented opposing results [30,31]. Despite a study showing that B cells were poorly permissive, they are susceptible to dengue viral infection, and the virus can directly induce polyclonal B cell activation [32]. Although dengue viral particles may play this role due to their presence in the blood circulation, viral proteins such as NS1 are also present in the blood during the acute phase of infection, with an observed high level in early viremia [33,34,35]. NS1 antigenemia has also been associated with more severe clinical symptoms [9,36]. Besides the importance of NS1 in the dengue viral life cycle, NS1 can disrupt the complement system [37], induce endothelial hyperpermeability [38] and activate cells via toll-like receptor 4 [39], suggesting multiple roles of NS1 during the course of dengue infection.

In this study, we suspected the role of NS1 in the induction of CD95 expression and apoptosis. Therefore, PBMCs from the healthy subjects were treated with recombinant NS1 protein at different concentrations, including 0.1, 0.5, 1 and 2 µg/mL [19], and observed for changes in CD95 and annexin V expression. The results showed that increased frequencies of cells expressing CD95 were observed in the naïve, tissue and activated memory B cell subsets when using the highest concentration of NS1 treatment (2 μg/mL), demonstrating the ability of the NS1 protein to induce the expression of CD95, especially in antigen-inexperienced naïve B cells. While only the highest concentration used in this study was able to increase the CD95 expression, the presence of NS1 at a similar level in the blood circulation of dengue-infected patients was previously demonstrated [19]. The study showed that the NS1 levels ranged from 0.04 to 2 μg/mL in the primary sera and from 0.01 to 2 μg/mL in the secondary sera, with up to 50 μg/mL able to be observed in one case [19], suggesting the possibility of in vivo CD95 induction by NS1 during acute dengue infection. The increased expression of CD95 in B cells may also contribute to the elevated soluble CD95 levels in the plasma in addition to its correlation with apoptotic CD8^+^ T lymphocytes, as previously observed during the course of dengue infection [40]. The capability of NS1 to induce early apoptosis in B cell subsets is also confirmed through the considerable levels of annexin V expression in all B cell subsets except for the resting memory B cells when treated with the same highest concentration of NS1. This finding is in compliance with a previous study reporting that B cells had an increased expression of active caspase-3, representing early apoptosis [8].

Regarding the role of the dengue viral NS1 protein in apoptotic induction, previous studies have shown that antibodies against NS1 cross-reacted with endothelial cells and induced apoptosis [15,17]. Moreover, the presence of anti-dengue virus NS1 antibody deposition and apoptotic cells on the liver vessel endothelium was observed in a murine model [41]. While anti-NS1 antibodies play a significant role in the mechanisms of endothelial cell apoptosis due to potential cross-reactivity to certain molecules with an ability to induce apoptosis, the influence of the NS1 protein on apoptosis may involve different pathways that can vary depending on the target cell. Although there is no evidence reporting that the NS1 protein directly induces apoptosis, the observation of NS1 inducing CD95 expression prompted us to investigate the role of NS1 in B cell apoptosis induction. Similar to the induction of CD95 expression, increased frequencies of apoptotic cells in all B cell subsets except for the resting memory B cells were observed when using the highest concentration of NS1 treatment, suggesting that NS1 can directly induce B cell apoptosis. More importantly, remarkably increased frequencies of apoptotic cells were shown in the naïve B cells and ASCs, suggesting that these subsets are more susceptible to apoptotic induction by NS1. The apoptotic induction mechanism, however, might not relate to the CD95 expression, since different levels of CD95 expression were observed in these B cell subsets. NS1 can modulate cell apoptosis by other alternative mechanisms, since a study has shown that NS1 can trigger endothelial cell apoptosis through pathways involving nitric oxide (NO) production [15]. Moreover, NS1 can also induce interleukin-10 (IL-10) production by monocytes, which is associated with T cell apoptosis [42].

It is crucial to acknowledge potential confounding factors, such as patient heterogeneity and systemic inflammation, that could influence the results obtained in this study. Since immune responses vary significantly with age, different age groups may exhibit different B cell responses to dengue infection. A history of previous dengue infections with different serotypes can also significantly impact the current immune response, as secondary infections often lead to more severe disease and altered B cell behavior. Moreover, dengue infection can trigger a cytokine storm, which is characterized by the excessive release of pro-inflammatory cytokines. These cytokines can directly influence B cell activation and CD95 expression. By carefully considering these potential confounders, future studies can gain a more accurate understanding of the role of CD95 expression in B cells in dengue infection.

## 5. Conclusions

This study demonstrates remarkably increased frequencies of naïve and resting memory B cell subsets expressing CD95 in parallel with decreased frequencies of these B cells in acute dengue-infected patients. The results also indicate that the increased expression of CD95 in B cells can be induced by the dengue viral NS1 protein. Moreover, B cell apoptosis can be directly induced by NS1. The findings thus suggest a potential mechanism for B cell subset alteration during the course of dengue infection due to the interaction of the dengue viral NS1 protein with the B cells, which directly induces cell apoptosis or indirectly increases CD95 expression, and the interaction with CD95L, causing cell apoptosis.

## Figures and Tables

**Table 1:** Numbers of recruited subjects according to their demographic data and characteristics.

Characteristic		DF	DHF	Healthy
Total subjects		10	12	26
Gender	Male	6	5	14
	Female	4	7	12
Age (years)		9–14	7–46	7–52
DENV serotype	1	1	2	-
	2	0	3	-
	3	5	1	-
	4	4	5	-
	1/4	0	1	-

## Data Availability

All relevant data are contained within the paper.
